# A Rare Cause of Abdominal Pain: Superior Mesenteric Artery Branch Pseudoaneurysm Rupture

**DOI:** 10.5152/tjg.2024.24170

**Published:** 2024-11-01

**Authors:** Qianqian Li, Yanhong Gao, Hao Yan, Chun Ye, Xingshun Qi

**Affiliations:** 1 Department of Gastroenterology, General Hospital of Northern Theater Command, Shenyang, China; 2 Department of Interventional Vascular Surgery, General Hospital of Northern Theater Command, Shenyang, China; 3 Department of General Surgery, General Hospital of Northern Theater Command, Shenyang, China

Dear Editor,

A 59-year-old female was admitted to our department due to progressive deterioration of abdominal pain for 1 week, accompanied by fever with the highest body temperature of 38°C on September 5, 2023. The abdominal pain radiated to the back. She had a history of mild acute pancreatitis 33 years ago, but the etiology of the acute pancreatitis was unclear. Physical examination showed right upper quadrant abdominal tenderness and percussion pain in the hepatic region without abdominal rebound tenderness. Laboratory tests showed mild anemia with a hemoglobin concentration of 107 g/L (reference range: 115-150 g/L) and elevated inflammatory indicators, but normal levels of serum amylase and lipase. Antibodies associated with vasculitis were negative. Unenhanced computed tomography (CT) scans showed abnormality in the region of the pancreatic head (Figure 1). Then, she underwent contrast-enhanced CT scans, which showed an irregularly shaped and high-density retroperitoneal lesion around the duodenum and pancreatic head. It had a CT value of 45 HU to 80 HU, suggesting a diagnosis of retroperitoneal hematoma (Figure 1). Thus, the abdominal pain was associated with compression of the retroperitoneal nerve plexus by the hematoma. On September 6, a CT-guided catheter was inserted to drain the hematoma and alleviate the pain. About 10 mL of dark red blood was drained, and the abdominal pain alleviated slightly. On September 7, gastroscopy and endoscopic ultrasound were performed to rule out the possibility that the retroperitoneal hematoma originated from gastric and duodenal stromal tumors. On the same day, CT angiography (CTA) scans were also performed, showing that a superior mesenteric artery branch was enlarged (Figure 1).

After a consultation with surgeons and vascular interventional radiologists, exploratory laparotomy was not the preferred choice due to the absence of active bleeding, intra-abdominal space-occupying lesions, or ruptured organs. In order to establish a definite diagnosis, percutaneous selective arteriography was performed after the patient and her son provided informed consent on September 11. The right femoral artery was punctured, and a 5F artery sheath was inserted. A catheter was delivered into the right hepatic artery through a guidewire. Ioversol extravasated locally from the middle segment of the superior mesenteric artery branch, suggesting a diagnosis of superior mesenteric artery branch pseudoaneurysm (SMAPA) (Figure 2). Coils were placed on both sides of the SMAPA to embolize the malformed artery. Neither the malformed artery nor the SMAPA was visible after reinjecting ioversol, and distal arteries were compensated by collateral circulation. Her abdominal pain disappeared without postoperative adverse reactions after embolization. Unenhanced CT scans performed afterward showed that the size of the retroperitoneal hematoma had decreased (Figure 1). Her hemoglobin level ranged from 96 g/L to 100 g/L. She was discharged without any complaints 10 days after the procedure. At her last visit on November 21, she did not feel any discomfort and had no anemia. The size of the retroperitoneal hematoma had decreased significantly on CT scans (Figure 1).

Our case highlighted the possibility of vascular-related diseases, including visceral artery pseudoaneurysm (VAPA), in patients with acute abdominal pain, to avoid a delay in treatment. Visceral artery pseudoaneurysm is rare but life-threatening due to the risk of its spontaneous rupture^1^. It can be located at the celiac artery, hepatic artery, splenic artery, superior mesenteric artery, inferior mesenteric artery and their branches. Among them, superior mesenteric artery branch pseudoaneurysm (SMAPA) is extremely rare. It is usually secondary to pancreatitis and rarely observed in trauma, iatrogenic injury, and other intra-abdominal inflammation.^1,2^ The rupture of SMAPA may occur in up to half of cases where the 3-layered vessel wall is incomplete.^3^ They often present with abdominal pain and massive hemorrhage, including hematemesis, hematochezia, or retroperitoneal hemorrhage.^1^

The imaging findings are very necessary for establishing a definite diagnosis. Visceral artery pseudoaneurysm appears as an anechoic structure on gray-scale Doppler images and a “yin-yang” configuration on color Doppler images. Although ultrasound is convenient and non-invasive, its accuracy is limited in diagnosing deep VAPA. Computed tomography angiography is commonly used in clinical practice because it can not only show a dilated artery, but also suggest the etiology and complications of VAPA.^1^ Angiography is the gold standard method for diagnosing vascular-related diseases. It is not the first-line choice of diagnosis due to its invasive nature but has an advantage of performing a therapeutic procedure at the same time.^4^ All patients with SMAPA are recommended to undergo timely treatment once they are diagnosed definitively.^4^ In the past, open surgery was often preferred for VAPA. It can repair the injured artery and resect organs, if necessary, but has a high risk of mortality.^1^ At present, endovascular procedures with the placement of a metallic coil or stent-graft have become the first-line choice of treatment because they are not only safe and effective but also suitable for patients in poor condition who cannot undergo open surgery, such as those with serious intra-abdominal inflammation and sepsis.^2,5^

## Figures and Tables

**Figure 1.**
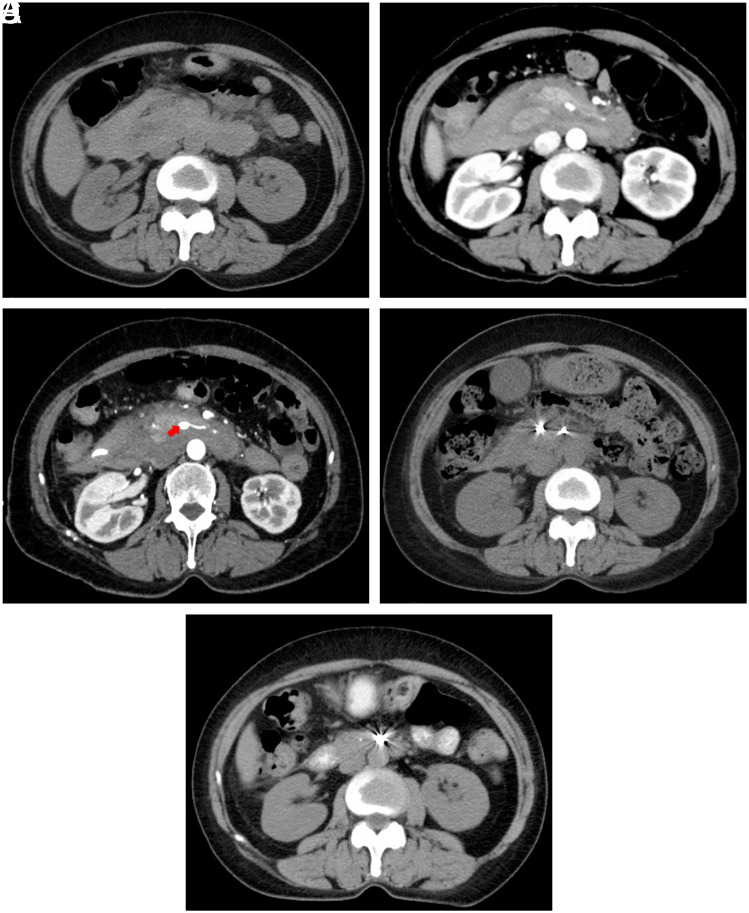
Change of this lesion on CT scans. (A) Irregularly shaped lesion around the pancreas on unenhanced CT scans. (B) Irregularly shaped and high-density retroperitoneal lesion on contrast-enhanced CT scans. (C) Enlargement of the superior mesenteric artery branch (red arrow) on CT angiography scans. (D and E) Radiation artifacts and a reduction in the size of the retroperitoneal hematoma at first and 10th weeks after endovascular treatment on unenhanced CT scans.

**Figure 2.**
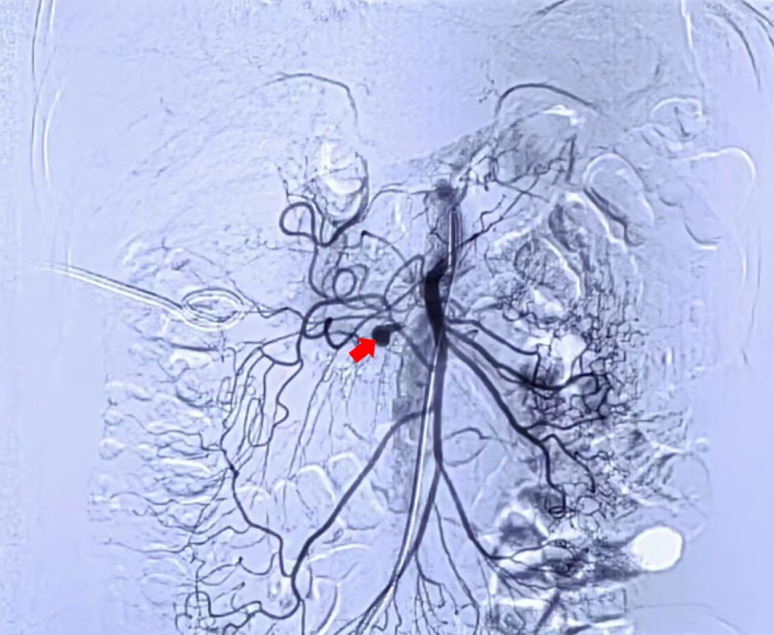
Cystic dilatation of the superior mesenteric artery branch (red arrow) on percutaneous selective arteriography.

## Data Availability

The data that support the findings of this study are available on request from the corresponding author.
